# Risk factors of postoperative delirium after cardiac surgery: a meta-analysis

**DOI:** 10.1186/s13019-021-01496-w

**Published:** 2021-04-26

**Authors:** Haiyan Chen, Liang Mo, Hongjuan Hu, Yulan Ou, Juan Luo

**Affiliations:** 1 Education and Training Department, The First Affiliated Hospital of University of South China, Hengyang, China; 2 Department of Cardiothoracic Surgery, The First Affiliated Hospital of University of South China, Hengyang, China; 3 Nursing Department, The First Affiliated Hospital of University of South China, Hengyang, China

**Keywords:** Postoperative delirium, Cardiac surgery, Meta-analysis

## Abstract

**Background:**

Postoperative delirium is a frequent event after cardiac surgery. This meta-analysis aimed to identify relevant risk factors.

**Method:**

In this meta-analysis, all original researches regarding patients undergoing mixed types of cardiac surgery (excluding transcatheter procedures) and postoperative delirium were evaluated for inclusion. On July 28th 2020, we searched PubMed, Embase, Web of Science and Scopus. Data about name of first author, year of publication, inclusion and exclusion criteria, research design, setting, method of delirium assessment, incidence of delirium, odds ratio (OR) and corresponding 95% confidence interval (CI) of risk factors, and other information relevant was collected. OR and 95% CI were used as metrics for summarized results. Random effects model was applied.

**Results:**

Fourteen reports were included with a total sample size of 13,286. The incidence of delirium ranged from 4.1 to 54.9%. Eight risk factors were identified including aging, diabetes, preoperative depression, mild cognitive impairment, carotid artery stenosis, NYHA functional class III or IV, time of mechanical ventilation and length of intensive care unit stay.

**Conclusion:**

In this study several risk factors associated with postoperative delirium after cardiac surgery were identified. Utilizing the information may allow us to identifying patients at high risk of developing postoperative delirium prior to delirium onset.

**Supplementary Information:**

The online version contains supplementary material available at 10.1186/s13019-021-01496-w.

## Background

The very first documented report regarding post-operative delirium after cardiac surgery can be dated back to 1964 [1]. During the following half century, the topic has been discussed thoroughly. Many risk factors have been identified, many countermeasures have been proposed [2], and many high-quality researches have been conducted. At this time, we believe it is necessary to look back and make a brief, yet focused, summary of this topic.

According to The American Psychiatric Association’s fifth edition of the *Diagnostic and Statistical Manual of Mental Disorders* (DSM-5), delirium is defined as a condition with following five key features: disturbance in attention and awareness; the disturbance develops over a short period of time and its severity tends to fluctuate during the course of a day; an additional disturbance in cognition; these mentioned disturbances cannot be better explained by other pre-existing neurocognitive disorders and do not occur in severely reduced arousal level such as coma; and there is evidence suggesting the disturbance is a direct result of another medical condition [3]. Delirium can be further classified into three sub-categories: hyperactive, hypoactive, and mixed. Patients with hyperactive delirium presents hypervigilance, agitation and restlessness, while hypoactive delirium is characterized by lethargy with decreased movement and slowed mental activity [4]. In practice, hypoactive delirium often remains unrecognized due to its difficulty of detection [5]. It has been reported that post-operative delirium within first few days after surgery is associated with worsened prognosis including prolonged hospitalization, increased mortality [6], cognitive impairment, memory decline, need for long-term care ^7^ and other unfavorable outcomes [4, 7, 8] in cardiac surgery [9] as well as other types of surgery, though it is still not clear whether delirium is a direct cause of [these outcomes or the association is mediated by other unidentified common cause of both delirium and the outcomes. Several pharmacologic or non-pharmacologic methods have been proposed to prevent delirium including early mobilization, improving Intensive Care Unit (ICU) environment, prophylactic antipsychotic administration, preoperative statins use [10], or use dexmedetomidine instead of propofol for sedation [11]. However, the reliability of these suggestions is undermined by lack of evidence, conflicting results, small sample sizes and imprecise delirium assessment [10, 12, 13].

Despite the effort made and the progress in surgical techniques during the past decades, the incidence of delirium after cardiac surgery remains between 26 and 52% when estimated with rigorous methodology [10], and the caring for delirious patients remains a major challenge in the daily work of cardiac surgical ICU nurses. Regular screening and early identification, together with appropriate sedation, are crucial in delirium management [14]. However, it would be ideal if we can recognize patients with high delirium risk in advance and make preparations accordingly. Koster et al. and Lin et al. have summarized the risk factors of delirium after cardiac surgery in their previous systemic review [15] and meta-analysis [16], in which 10 and 25 original studies were included, leading to the revealment of a total of 27 and 33 risk factors, respectively. In the systemic review by Koster et al., 10 publications within the period January 1999 through December 2009 were reviewed, with 27 risk factors identified, while only 10 of them were mentioned more than once. Meta-analysis was not performed Koster et al., making the summarization merely qualitative. In the meta-analysis by Lin et al., which included literatures from 2008 to 2011, age increase, depression, stroke history and several other risk factors were identified, while the mixed use of univariate/multivariate results and RR/OR values made the conclusions less robust mathematically. Besides, recent studies in which POD was diagnosed with Diagnostic and Statistical Manual of Mental Disorders Fifth Edition (DSM-V) were not analyzed by Koster et al. or Lin et al., suggesting the necessity of an updated summarization including original studies emerged from the most recent decade, given the rapid evolvement of clinical practice. The objective of this meta-analysis was to identify risk factors of delirium in patients undergoing mixed types of cardiac surgery excluding transcatheter procedures.

## Method

### Search strategy and inclusion criteria

To ensure the quality of this meta-analysis, inclusion criteria was decided before we carried out the search. These criteria were:

1. Only original researches are eligible for inclusion. Reviews, meta-analyses, study protocols, comments, editorials and errata shall not be considered for analysis.
2. Eligible studies must be carried out in cohorts containing more than one adult (> 18 years old) patient undergoing electiveopen-heart surgery, which was defined as any type of surgery in which an incision is made on chest and procedure is done on coronary artery, heart valve or myocardium, due to non-congenital heart disease.
3. Delirium must be assessed with one of the following tools: Diagnostic and Statistical Manual of Mental Disorders, Fourth or Fifth Edition (DSM-IV or DSM-V), Confusion Assessment Method (CAM), Confusion Assessment Method for the ICU (CAM-ICU), Intensive Care Delirium Screening Checklist (ICDSC) or other well-validated diagnostic standard based on the tools mentioned above. Researches focused on only one subtype of delirium (hyperactive delirium, hypoactive delirium or mixed delirium) shall not be included since the above tools recognize delirium of all subtypes but does not provide differential diagnosis among subtypes.
4. Risk factors for delirium must be assessed with odds ratio (OR) with 95% confidence interval (CI). A research must present the results of both univariate and multivariate regression to be considered eligible for inclusion, since multivariate analysis results shall be used to identify variables eligible for meta-analysis and the actual meta-analysis shall be conducted on univariate analysis results.
5. Only full-text available literatures in English language are included.
6. For multiple reports on overlapping population, only the study with largest sample size shall be included to avoid including the same participants multiple times.

On July 28th 2020, a search was conducted on PubMed, Embase, Web of Science and Scopus in all past publications with following keywords: “delirium” AND (“cardiac surgery” OR “heart surgery” OR “coronary artery bypass grafting” OR “cardiopulmonary bypass” OR “valve surgery”) AND “risk factor*”. The search yielded 268 results on PubMed, 559 results on Embase, 369 results on Web of Science and 448 results on Scopus. After removing duplicate entries automatically with Mendeley, 935 entries were screened by title, abstract or full text to identify eligible literatures. The screening process was independently performed by two investigators and discrepancies were resolved through discussion with a third reviewer.

### Data extraction and analysis

Data extracted from selected literatures included information of authors, year of publication, sample size, inclusion and exclusion criteria, baseline features of patients, research design, research setting, method of delirium assessment, odds ratio (OR) and corresponding 95% confidence interval (CI) of risk factors, *p* value, and other information considered relevant. A risk factor is eligible for meta-analysis if (1) it is identified as independent predictor in multivariate regression in at least one of included literatures; (2) its OR and corresponding 95% confidence interval in univariate regression are reported in at least two of included literatures. We used only the OR and CI of risk factors resulted from univariate regression for analysis: a statement of statistical methodology applied was provided in Additional file 1. The identified risk factors shall be reported as preoperative, intraoperative and postoperative risk factors, since the traditional classification of predisposing and precipitating risk factors cannot be precisely recognized, mainly because of the complexity identifying the direct cause of POD. Thus, we chose a more practical classification to allow the results of this meta-analysis to be easily applied in clinical practice, facilitating the identification of patients at higher risk of POD.

Statistical analysis was conducted with meta package [17] in R. Between-study variance was evaluated with DerSimonian-Laird estimator. Heterogeneity among studies was measured with Cochran chi-square test and was considered present if *p* value is less than 0.1. Heterogeneity statistic I^2^ was used to indicate level of heterogeneity. When I^2^ was lower than 25%, studies analyzed were considered of low heterogeneity, while an I^2^ value greater than 50% indicated the presence of high heterogeneity. Pooled odds ratios and 95% confidence intervals were evaluated with both fixed and random effects models; however, all conclusions of this meta-analysis were drawn from the random effects model results. Fixed effect analysis was done for reference only. For meta-analyses containing more than four studies, sensitivity analysis, in which studies were omitted once at a time to evaluate their impact to the final pooled result, and funnel plot analysis were performed.

### Risk of Bias and quality of evidence assessment

Risk of bias assessment for each included study was performed using Newcastle-Ottawa Scale by two reviewers, independently. A third reviewer shall be invited to discussion to achieve consensus in case of disagreement [18]. Case-control studies were evaluated regarding case and control selection (4 stars), comparability between cases and controls (2 stars), and assessment of exposure (3 stars); while cohort studies were evaluated regarding selection of exposed and control cohorts (4 stars), comparability between cohorts (2 stars), and assessment of outcome (3 stars). Studies were considered to be at low risk if rated 7 stars or above, moderate risk if rated 4–6 stars, and high risk if less than 4 stars.

Quality of evidence assessment was performed through discussion among three reviewers using Grading of Recommendations, Assessment, Development and Evaluations (GRADE). For each result of this meta-analysis, one of the following ratings was assigned: High, where authors were confident that estimated effect was similar to the true effect; Moderate, where estimated effect was probably close to the true effect; Low, where the true effect might be different from the estimated effect; and Very low, where the true effect was probably markedly different from the estimated effect.

## Result

### Summary

**Fig. 1:**
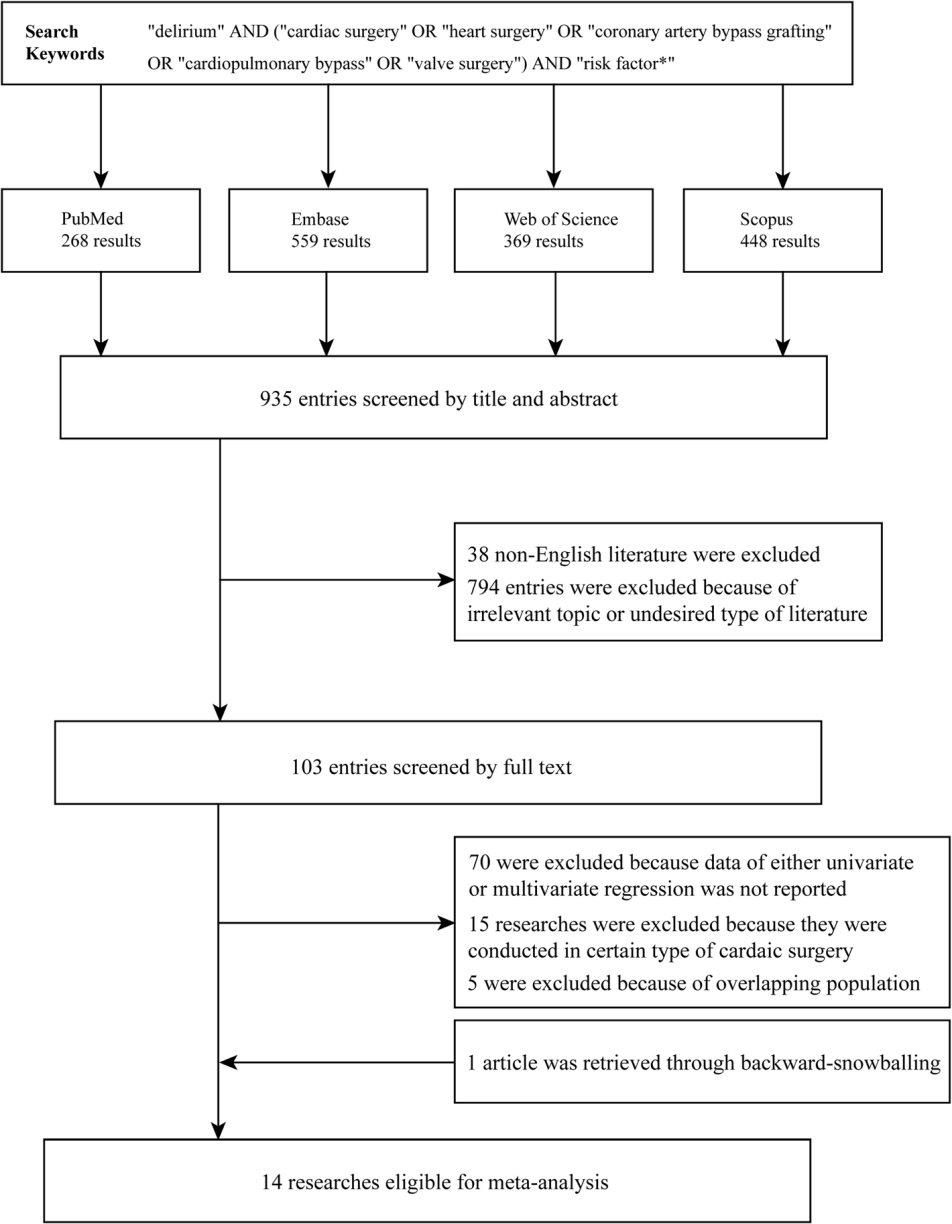
The flowchart of literature selection process

A total of 14 studies met our inclusion criteria and was included in this meta-analysis [19–32]. The selection process was summarized with a flowchart (Fig. 1). The main features of these studies were summarized in Table 1. In summary, the studies eligible contained 7 prospective cohort studies, 4 prospective observational studies and 3 retrospective analyses, the sum of sample size being 13,286. The incidence of developing postoperative delirium (POD) ranged from 4.1 to 54.9%.

**Table 1:** Main features of studies included

Study Included	Design	Setting	Sample Size	Incidence	Diagnosis Standard	Risk Factors Identified
Burkhart 2010 [19]	Retrospective analysis	University Hospital Basel, Basel, Switzerland	113	30%	CAM	Max CRP, Fentanyl intraoperatively, Duration of mechanical ventilation
Cai 2020 [20]	Prospective cohort study	Zhongshan Hospital, Shanghai, China	635	11.5%	CAM-ICU	NT-proBNP, NYHA functional classification III or IV, LVEF
Itagaki 2020 [21]	Retrospective analysis	The Cardiovascular Institute, Tokyo, Japan	89	28.1%	ICDSC	Age, MCI (MoCA< 26 points), Frailty (J-CHS3 points)
Katznelson 2009 [22]	Prospective cohort study	Toronto General Hospital, Toronto, Canada	1059	11.5%	CAM-ICU	Red blood cell transfusion, (> 5 units), Perioperative intra-aortic balloon pump support, Preoperative depression, Preoperative creatinine > 150 mM, Age ≥ 60 years, Combined CABG and valvular surgery. Preoperative administration of statins was found protective
Kazmierski 2010 [23]	Prospective cohort study	WAM University Hospital in Lodz, Lodz, Poland	563	16.3%	DSM-IV	Age ≥ 65 years, MMSE < 25, Major depression, Anemia, AF, Intubation > 24 h, pO2 < 60 mmHg
Kotfis 2019 [24]	Prospective cohort study	Hospital of Pomeranian Medical University, Szczecin, Poland	3178	15.8%	DSM-V	Age, NYHA class III or IV, HbA1c, Creatinine at admission, Extracardiac arteriopathy
Krzych 2013 [25]	Prospective observational study	Upper Silesia Medical Center, Katowice, Poland	5781	4.1%	DSM-IV	Postoperative cerebral ischemia, blood transfusion, Age > 65 years, Carotid artery stenosis, Emergency surgery, Fasting glucose level, ΔpO2, ΔHematocrit. Hypertension was found protective
Norkiene 2013 [26]	Prospective cohort study	Vilnius University Hospital, Vilnius, Lithuania	87	13.30%	ICDSC	ICU stay, Duration of controlled mechanical ventilation
Ogawa 2018 [27]	Prospective cohort study	Kobe University Hospital, Kobe, Japan	313	14.6%	ICDSC	6MWD, ICU stay
Sabol 2015 [28]	Prospective observational study	VUSCH, Kosice, Slovakia	250	20.8%	CAM-ICU	Age, EuroSCORE II, CPB time, ACC time, Sufentanil dose, Benzodiazepine administration, CABG
Sauër 2017 [29]	Retrospective analysis	University Medical Center Utrecht, Utrecht, the Netherlands	184	12.5%	CAM-ICU	Trailmaking test A
Smulter 2013 [30]	Prospective observational study	Umeå University Hospital, Umeå, Sweden	142	54.9%	DSM-IV	NRS pain, Diabetes, Oxygen saturation peripheral, Combined surgical procedure, Volume load (blood excluded), Ventilator time, Sodium concentration in ICU
Tully 2010 [31]	Prospective cohort study	Flinders Medical Centre, Adelaide, Australia	158	31%	Delirium Symptom Interview (DSI) based on DSM-IV	ACC time, hemoglobin, Major depressive disorder, Psychotropic/anti-cholinergic drug use
Wesselink 2015 [32]	Prospective observational study	University Medical Center Utrecht, Utrecht, the Netherlands	734	13%	CAM and CAM-ICU	MAP< 60 mmHg

*6MWD* 6-min walking distance, *ACC* aortic cross-clamping, *AF* atrial fibrillation, *CABG* coronary artery bypass grafting, *CAM* Confusion Assessment Method, *CPB* cardiopulmonary bypass, *HbA1c* glycated hemoglobin, *LVEF* left ventricular ejection fraction, *DSM* Diagnostic and Statistical Manual of Mental Disorders, *ICU* intensive care unit, *ICDSC* Intensive Care Delirium Screening Checklist, *MAP* mean arterial pressure, *Max CRP* maximum value of C-reactive protein postoperatively, *MCI* mild cognitive impairment, *MMSE* Mini-Mental State Examination, *MoCA* Montreal Cognitive Assessment, *NRS* Numeric Rating Scale, *NT-proBNP* N-terminal prohormone of brain natriuretic peptide, *NYHA* New York Heart Association, *pO2* arterial oxygen partial pressure. Δ: intraoperative fluctuation (max-min)

### Risk of Bias and quality of evidence assessment

Risk of bias assessment for eligible studies was summarized in Additional file 2, where all included studies were considered to be of low risk methodologically according to the Newcastle-Ottawa Scale. Quality of evidence assessment results using GRADE were presented in Additional file 3, in which GRADE certainty for meta-analysis result for variable hypertension was rated as moderate due to conflicting results of included studies, and the other results were considered to be of high confidence [33].

### Preoperative risk factors

In this category, 9 variables were eligible for meta-analysis, including age (increase by year or > 65 years), carotid artery stenosis, diabetes, hypertension, left ventricular ejection fraction (LVEF) percentage, preoperative depression, New York Heart Association (NYHA) functional classification III or IV, mild cognitive impairment (MCI), and preoperative statins use.

**Fig. 2:**
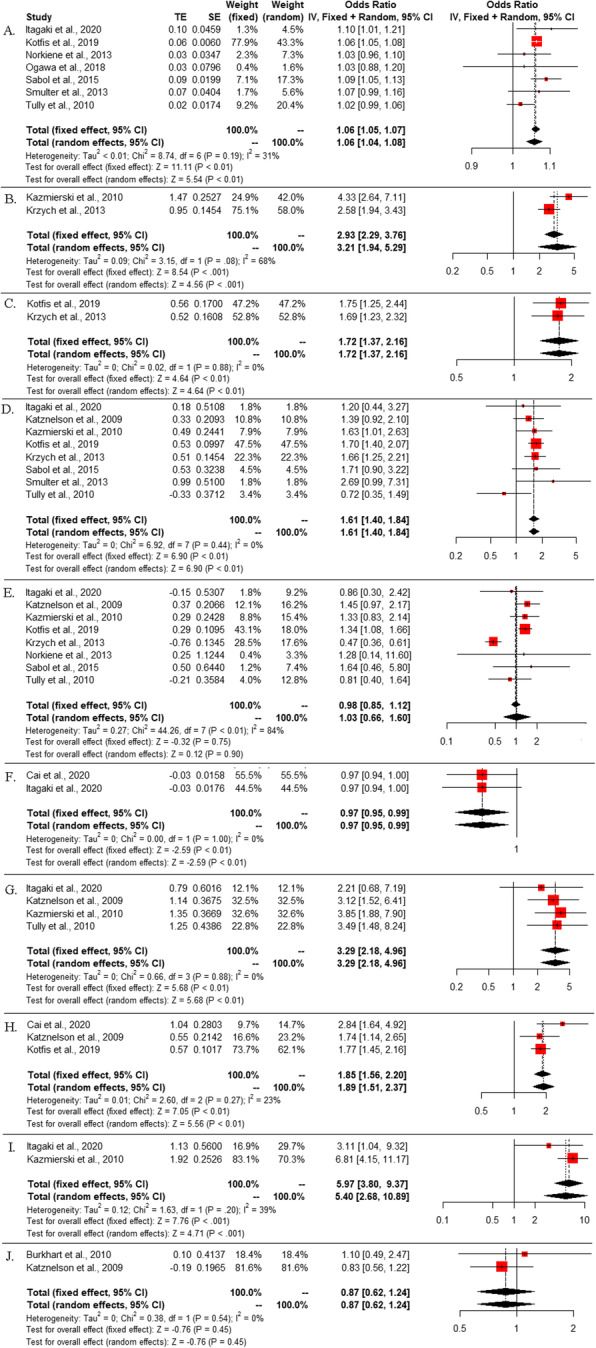
The forest plots summarizing preoperative risk factors. **a** Age increase per year. **b** Age > 65 years. **c** Carotid artery stenosis. **d** Diabetes. **e** Hypertension. **f** Left ventricular ejection fraction by percentage. **g** Preoperative depression. **h** NYHA functional class III or IV. **i** Preoperative mild cognitive impairment. **j** Preoperative use of statins. Results were summarized with OR and corresponding 95% CI. I2 statistic was used to assess heterogeneity among studies

Aging was identified as risk factor by nine researches, of which seven reported higher risk of POD when patients’ age increased by year [21, 24, 26–28, 30, 31], while other two found age > 65 was significantly associated with increased POD risk. Meta-analysis of these studies revealed significant increase of POD risk as patients’ age increased. (OR = 1.06, 95% CI: [1.04, 1.08], I^2^ = 31% for age increase per year; OR = 3.21, 95% CI: [1.94, 5.29], I^2^ = 68% if patients older than 65) (Fig. 2a, b).

Carotid artery stenosis was identified as risk factor by three researches [24–26], however, throughout inspection suggested there might be a mistake when reporting relevant data in one article as the data presented in table was exactly the same with its next row [26], so only two reports were eligible for meta-analysis, showing significantly increased risk in patients with carotid artery stenosis (OR = 1.72, 95% CI: [1.37, 2.16], I^2^ = 0%) (Fig. 2c).

Diabetes-related POD OR data in univariate regression was reported in eight studies [21–25, 28, 30, 31]. Meta-analysis of these report showed significantly increased OR in patients with diabetes (OR = 1.61, 95% CI: [1.40, 1.84], I^2^ = 0%) (Fig. 2d).

Hypertension was found associated with lower OR of POD in one study [25], while among other studies the relationship was insignificant [21–26, 28, 31]. The result of our meta-analysis supported the latter conclusion (OR = 1.03, 95% CI: [0.66, 1.60], I^2^ = 84%) (Fig. 2e).

Heart-function related risk factors were also identified within eligible literature. LVEF increase by percentage was found to be protective by Cai et al. [20], and was supported by our meta-analysis together with data reported by Itagaki et al. [21] (OR = 0.97, 95% CI: [0.95, 0.99], I^2^ = 0%) (Fig. 2f), while three researches reported NYHA functional classification III or IV was associated with significantly increased OR of POD [20, 22, 24] (OR = 1.89, 95% CI: [1.51, 2.37], I^2^ = 23%) (Fig. 2h).

Preoperative depression was identified as risk factor in three studies [22, 23, 31]. After combining the three studies with data provided by another report [21], meta-analysis showed patients with preoperative depression suffered from higher risk of POD (OR = 3.29, 95% CI: [2.18, 4.96], I^2^ = 0%) (Fig. 2g). Preoperative mild cognitive impairment was identified as risk factor in two studies [21, 23], where the term was defined as Montreal Cognitive Assessment (MoCA) < 26 or Mini-Mental State Examination (MMSE) < 25, respectively. Our meta-analysis of these two studies showed preoperative cognitive impairment was associated with increased risk of POD (OR = 5.40, 95% CI: [2.68, 10.89], I^2^ = 39%) (Fig. 2i).

Preoperative use of statin was found to be a protective factor by Katznelson et al. [22]. However, meta-analysis combining data from another report [19] failed to support the conclusion (OR = 0.87, 95% CI: [0.62, 1.24], I^2^ = 0%) (Fig. 2j).

### Intraoperative and postoperative risk factors

In this category, 3 variables were eligible for meta-analysis, including aortic cross clamp (ACC) time (per minute), intensive care unit (ICU) stay (per day) and mechanical ventilation time (per hour).

**Fig. 3:**
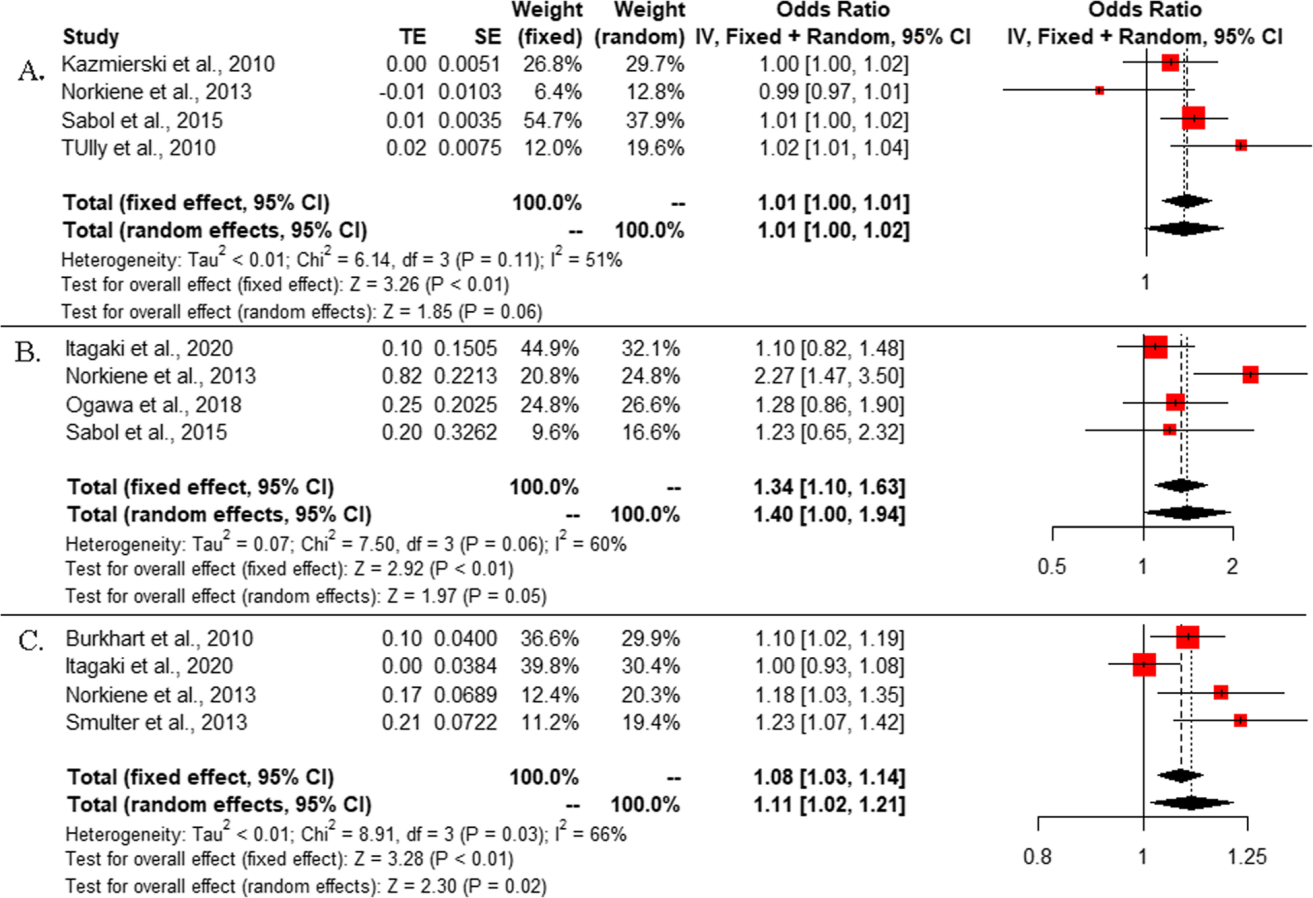
The forest plots summarizing intra- and postoperative risk factors. **a** Aortic cross clamp time per minute. **b** ICU stay per day. **c** Mechanical ventilation time per hour. Results were summarized with OR and corresponding 95% CI. I2 statistic was used to assess heterogeneity among studies

ACC time related POD OR data was reported in four researches [23, 26, 28, 31]. Pooled analysis showed that for every one-minute increase in ACC time, the OR for POD increased by 1% (OR = 1.01, 95% CI: [1.00, 1.02], I^2^ = 51%) (Fig. 3a). However, in test for overall effect the variable failed to achieve statistical significance (*p* = 0.06).

Two researches identified length of ICU stay was associated with increased OR of POD [26, 27]. When combined with data from other two studies [21, 28], meta-analysis supported the conclusion, showing for every one more day of ICU stay was associated with 40% increase OR of POD (OR = 1.40, 95% CI: [1.00, 1.94], I^2^ = 60%) (Fig. 3b). Finally, meta-analysis using data from four studies also showed increased time of mechanical ventilation (per hour) was associated with higher risk of POD [19, 21, 26, 30] (OR = 1.11, 95% CI: [1.02, 1.21], I^2^ = 66%) (Fig. 3c).

### Sensitivity analysis and funnel plot analysis

**Fig. 4:**
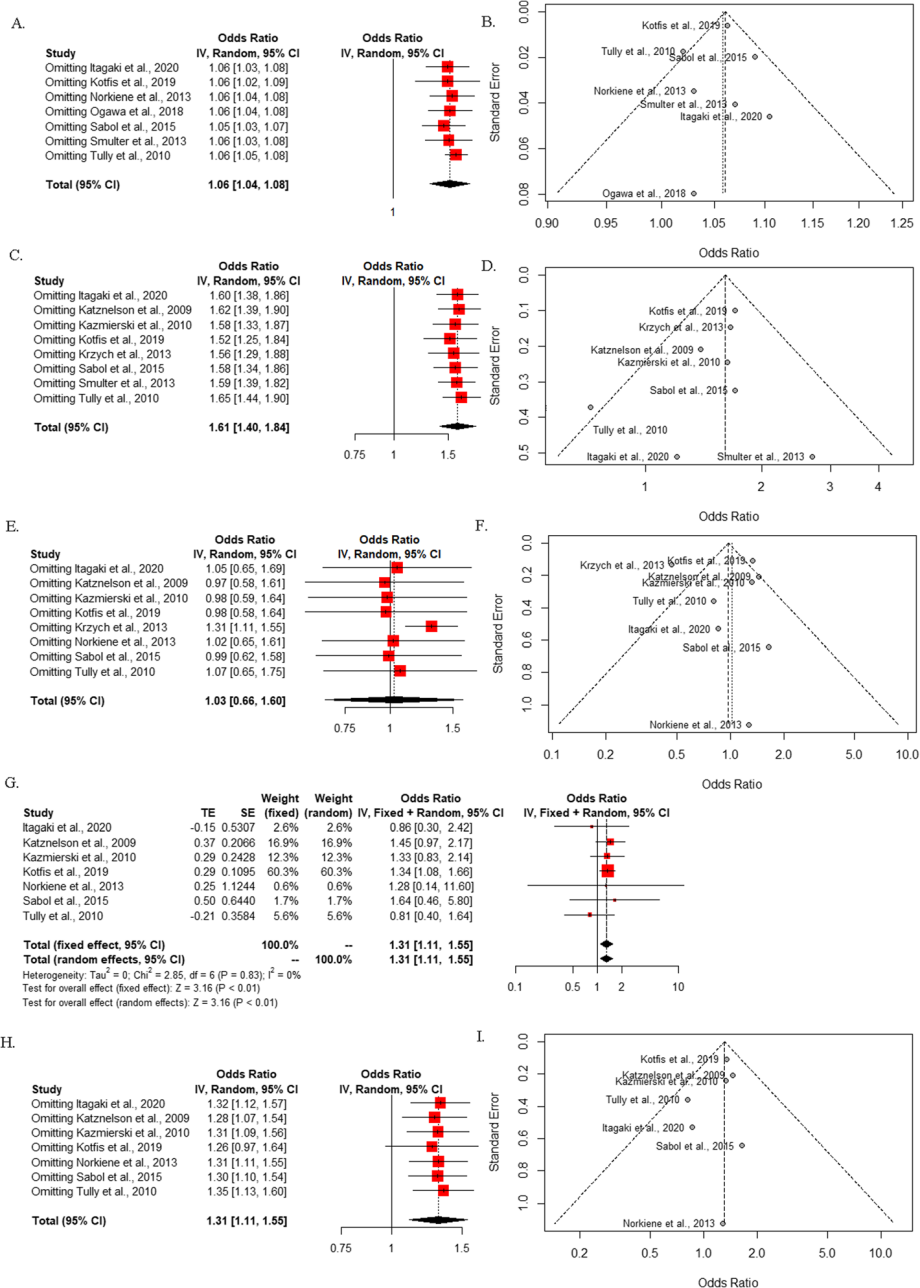
Sensitivity analysis and funnel plot of analyses containing more than 4 studies. **a** Sensitivity analysis for age increase per year. **b** Funnel plot for age increase per year. **c** Sensitivity analysis for diabetes. **d** Funnel plot for diabetes. **e** Sensitivity analysis for hypertension. **f** Funnel plot for hypertension. **g** Forest plot of hypertension as risk factor after excluding the study that had major impact to pooled result. **h** Sensitivity analysis for hypertension after excluding the study that had major impact to pooled result. **i** Funnel plot for hypertension after excluding the study that had major impact to pooled result

In Meta-analyses regarding age (per year), diabetes and hypertension as risk factors for POD after cardiac surgery, sensitivity analysis and funnel plot analysis was performed. In the age (per year) analysis, sensitivity analysis showed no individual research had major impact on final pooled result (Fig. 4a), however, funnel plot analysis revealed potential asymmetry (Fig. 4b). In the diabetes analysis, sensitivity analysis returned similar result (Fig. 4c) and funnel plot was of good symmetry (Fig. 4d). In the hypertension analysis, funnel plot analysis revealed the presence of asymmetry (Fig. 4f), and sensitivity analysis showed the study by Krzych et al. had a major impact on result of meta-analysis [25] (Fig. 4e).

### Post-hoc analysis

To evaluate the impact of the study by Krzych et al. on the meta-analysis of hypertension variable, a post-hoc analysis was conducted by excluding this study from the analysis and a new forest plot was formed. The post-hoc analysis suggested hypertension was associated with increased risk of POD after cardiac surgery (OR = 1.31, 95% CI: [1.12, 1.55], I^2^ = 0%) (Fig. 4g). After excluding the study, sensitivity analysis showed stable result (Fig. 4h) and funnel plot symmetry was restored (Fig. 4i). We also noted that after excluding the study, I^2^ statistic measuring heterogeneity turned to 0%, compared to the previous value of 84%, suggesting the study by Krzych et al. was the primary cause of heterogeneity.

## Discussion

To summarize, through this meta-analysis, we identified a series of risk factors for POD after cardiac surgery including older age, carotid artery stenosis, diabetes, preoperative depression or mild cognitive impairment, NYHA functional class III or IV, length of ICU-stay or mechanical ventilation. We also identified LVEF as a protective factor, and preoperative statins use not significantly associated with development of POD. We hope the information provided by our meta-analysis can be used to assist the daily nursing practice of cardiac surgery ICU, allowing patients with high risk of developing delirium to be identified in advance.

In our study, all risk factors were empirically classified into preoperative, intraoperative and postoperative risk factors. The aim of doing so was to make the result of this study easier utilized in practical nursing work. Surprisingly we found that among preoperative analyses, the heterogeneity among studies was generally lower than the heterogeneity of intra- and postoperative risk factors, which could potentially be explained as preoperative risk factors have similar effect in general population while the effects of intra-and postoperative risk factors are different due to the difference in medical practice among different hospitals. We also noted these identified risk factors fell into four partly overlapping groups: risk factors associated with general condition such as age and diabetes; ischemic condition related risk factors such as carotid artery stenosis, NYHA III or IV and LVEF; cognition-related risk factors such as preoperative depression and mild cognitive impairment; and intervention related risk factors such as mechanical ventilation time and length of ICU stay. These features may enlighten future research in POD etiology. However, it is important to note that none of these identified risk factors can be declared to be causative at present since the mechanism and direct cause of POD remains to be determined.

In order to interpret the results of our meta-analysis in context of other evidence, we conduct a search on PubMed with the keywords “delirium cardiac surgery meta-analysis”. In the study by Vallabhajosyula et al. evaluating the effect of statins on POD prevention, meta-analysis of 6 included studies with 4382 patients underwent cardiac surgery (and statins were used in 2321 of them) concluded that statin therapy did not benefit delirium prevention, which was consistent with our results [13]. In the systemic review by Greaves et al. specifically summarize risk factors for POD following coronary artery bypass grafting surgery, cognitive impairment was found to show the largest effect, followed by stroke history and depression. Diabetes and hypertension were also significantly associated with higher risk of POD [34]. In transcatheter aortic valve implantation, risk factors for POD identified by Tilley et al. included coronary artery disease and hypertension, while NYHA III-IV, LVEF, age and diabetes were not statistically significant [35]. The partly-overlapping conclusions of these studies, including our meta-analysis, strongly suggested not only existence of association between the identified risk factors and POD but also the difference in nature of distinct surgery types.

For hypertension variable, the initial meta-analysis revealed no statistically significant association between the variable and POD occurrence. However, when the forest plot and corresponding funnel plot were inspected by the authors, we found the study by Krzych et al. may have a major impact to the result of analysis, as indicated by the leave-one-out sensitivity analysis, and caused asymmetry in funnel plot. A post-hoc analysis was decided to investigate if there was an association between hypertension and POD occurrence when the study by Krzych et al. was excluded. Interestingly hypertension was found to be a significant risk factor in the post-hoc analysis (OR = 1.31, 95% CI: [1.12, 1.55], I^2^ = 0%) (Fig. 4g). We believed the impact of Krzych study was because of its relatively large sample size and conflicting direction of effect. Besides, potential funnel plot asymmetry was also noticed in analysis of hypertension variable. We believed the presence of small-study effect might not be a satisfying explanation since all studies except the Krzych 2013 study located somewhere near the line of symmetry of funnel plot. Generally, study with lower standard error gives more accurate estimation for effect, while in this analysis a major disagreement among large studies presented. The inclusion of Krzych study on hypertension variable made the non-significant result of the initial analysis less robust, while result from the post-hoc analysis needed to be interpreted with more caution since the exclusion was only based on sensitivity analysis and funnel plot imbalance, when the study was rated of low risk of bias methodologically (8 stars with Newcastle-Ottawa Scale), resulting in a lower level of evidence of the post-hoc analysis result. Here we chose not to report hypertension as a risk factor for POD and believed further research is needed regarding this question.

Finally, it is important to specify potential limitations of this study. Due to the strict inclusion criteria, only a few reports were considered eligible for analysis, making this study at risk of omitting potentially valuable information. However, due to the mentioned reason, we believed it was a necessary sacrifice and hereby strongly advice researchers to present both the result of univariate and multivariate regression. Second, the risk factors identified might contain confounding factors. For example, length of ICU stay could be a confounder of mechanical ventilation time, dose of sedative medication and so on, while these variables were left unadjusted. Third, considering the retrospective nature of certain included studies and dropouts in prospective cohort studies, selection bias might present. Fourth, the diagnosis standard of comorbidities such as hypertension, diabetes and preoperative depression might be not same among individual studies.

## Conclusion

In conclusion, in this meta-analysis, 8 risk factors and 1 protective factor were identified in 14 researches. Further research remains necessary to validate these conclusions and to investigate the underlying mechanisms.

## Supplementary Information

**Additional file 1.** Supplementary

**Additional file 2.** Risk of bias assessment for included studies using Newcastle-Ottawa Scale. Studies were considered to be at low risk if rated 7 stars or above, moderate risk if rated 4–6 stars, and high risk if less than 4 stars.

**Additional file 3.** Level of evidence assessment of meta-analysis results using Grading of Recommendations, Assessment, Development and Evaluations (GRADE).

## Data Availability

All data generated or analysed during this study are included in this published article and its supplementary information files.

## Abbreviations

ACC: Aortic cross clamp
CAM: Confusion Assessment Method
CI: Confidence interval
DSM: Diagnostic and Statistical Manual of Mental Disorders
ICDSC: Intensive Care Delirium Screening Checklist
ICU: Intensive Care Unit
LVEF: Left ventricular ejection fraction
MCI: Mild cognitive impairment
MMSE: Mini-Mental State Examination
MoCA: Montreal Cognitive Assessment
NYHA: New York Heart Association
OR: Odds ratio
POD: Postoperative delirium
