# Supplementary material for: Risk factors of postoperative delirium after cardiac surgery: a meta-analysis
Source: J Cardiothorac Surg. 2021 Apr 26;16:113. doi: 10.1186/s13019-021-01496-w (PMC8072735; doi:10.1186/s13019-021-01496-w)
Supplement: Supplementary file 1 — Additional file 1. Supplementary [file 13019_2021_1496_MOESM1_ESM.docx]

**Supplementary**

In this study, we identified candidate risk factors from multivariate regression models from each study, however, the meta-analysis was carried out based on the odds ratios and confidence intervals provided by univariate regression models from individual study. It is important to justify the approach. In brief, the approach is more conservative and conserved information from original research as much as possible. The statement was made based on the following fact that in practical work researches tend to identify potentially valuable variables with univariate regression, then construct a multivariate regression model with such variables, meaning multivariate regression models usually contain much less variables and usually only ones significant related with the defined outcome in univariate regression models. Consider an example in which 6 independent researchers were investigating a same topic: evaluating the effect of a series of risk factors on a certain outcome D, and they all had same patient sample size. For simplicity, let’s assume none of these factors had any actual effect on D. However, due to random error, selection bias or any other reasons, every combination of 2 researchers found a shared spurious relationship and the “risk factor” was used to construct multivariate regression models, while in other 4 researchers the “risk factor” was found to be insignificant and was thus left in univariate regression models. A complete combination could produce 12 of such spurious “risk factors”. At this moment, if a meta-analysis only looked into the parameters of multivariate regression model, it would yield a combination of such 12 “risk factors”, making the meta-analysis a combination of fallacies. However, if the meta-analysis was based on results of univariate models, the information provided by all 6 researches could be included and the true relationship (not associated) between these factors and outcome D would potentially be revealed. Combining the results from both univariate and multivariate regression models was not feasible since the meaning of the approach cannot be justified mathematically.

This method was used by meta-analysis researchers to avoid the biased introduced by variability in methods and variables used to derive the final multivariate model and to improve across study comparability [1-4].

**Reference**

1. McCormick Matthews LH, Noble F, Tod J, Jaynes E, Harris S, Primrose JN, et al. Systematic review and meta-analysis of immunohistochemical prognostic biomarkers in resected oesophageal adenocarcinoma. British journal of cancer. 2015;113(1):107-18; doi: 10.1038/bjc.2015.179.

2. Kamiya H, Panlaqui OM. Systematic review and meta-analysis of prognostic factors of acute exacerbation of idiopathic pulmonary fibrosis. BMJ open. 2020;10(6):e035420; doi: 10.1136/bmjopen-2019-035420.

3. van Laar M, van Amsterdam WAC, van Lindert ASR, de Jong PA, Verhoeff JJC. Prognostic factors for overall survival of stage III non-small cell lung cancer patients on computed tomography: A systematic review and meta-analysis. Radiother Oncol. 2020;151:152-75; doi: 10.1016/j.radonc.2020.07.030.

4. Greaves D, Psaltis PJ, Davis DHJ, Ross TJ, Ghezzi ES, Lampit A, et al. Risk Factors for Delirium and Cognitive Decline Following Coronary Artery Bypass Grafting Surgery: A Systematic Review and Meta-Analysis. J Am Heart Assoc. 2020;9(22):e017275; doi: 10.1161/JAHA.120.017275.
