# Supplementary material for: Risk factors of postoperative delirium after cardiac surgery: a meta-analysis
Source: J Cardiothorac Surg. 2021 Apr 26;16:113. doi: 10.1186/s13019-021-01496-w (PMC8072735; doi:10.1186/s13019-021-01496-w)
Supplement: Supplementary file 2 — Additional file 2. Risk of bias assessment for included studies using Newcastle-Ottawa Scale. Studies were considered to be at low risk if rated 7 stars or above, moderate risk if rated 4–6 stars, and high risk if less than 4 stars. [file 13019_2021_1496_MOESM2_ESM.docx]

**Additional file 2.** Risk of bias assessment for included studies using Newcastle-Ottawa Scale. Studies were considered to be at low risk if rated 7 stars or above, moderate risk if rated 4-6 stars, and high risk if less than 4 stars.

| **Study Included** | Selection  (4 stars) | Comparability  (2 stars) | Exposure/Outcome  (3 stars) | Total  (9 stars) |
| --- | --- | --- | --- | --- |
| Burkhart 2010 [19] | ★★★ | ★★ | ★★★ | 8 |
| Cai 2020 [20] | ★★★ | ★★ | ★★★ | 8 |
| Itagaki 2020 [21] | ★★★★ | ★★ | ★★★ | 9 |
| Katznelson 2009 [22] | ★★★ | ★★ | ★★★ | 8 |
| Kazmierski 2010 [23] | ★★ | ★★ | ★★★ | 7 |
| Kotfis 2019 [24] | ★★★ | ★★ | ★★★ | 8 |
| Krzych 2013 [25] | ★★★ | ★★ | ★★★ | 8 |
| Norkiene 2013 [26] | ★★★ | ★★ | ★★★ | 8 |
| Ogawa 2018 [27] | ★★★ | ★★ | ★★★ | 8 |
| Sabol 2015 [28] | ★★★ | ★★ | ★★★ | 8 |
| Sauër 2017 [29] | ★★ | ★★ | ★★★ | 7 |
| Smulter 2013 [30] | ★★★ | ★★ | ★★★ | 8 |
| Tully 2010 [31] | ★★★ | ★★ | ★★★ | 8 |
| Wesselink 2015 [32] | ★★ | ★★ | ★★★ | 7 |
