# Supplementary material for: Risk factors of postoperative delirium after cardiac surgery: a meta-analysis
Source: J Cardiothorac Surg. 2021 Apr 26;16:113. doi: 10.1186/s13019-021-01496-w (PMC8072735; doi:10.1186/s13019-021-01496-w)
Supplement: Supplementary file 3 — Additional file 3. Level of evidence assessment of meta-analysis results using Grading of Recommendations, Assessment, Development and Evaluations (GRADE). [file 13019_2021_1496_MOESM3_ESM.docx]

**Additional file 3.** Level of evidence assessment of meta-analysis results using Grading of Recommendations, Assessment, Development and Evaluations (GRADE).

| Variable | GRADE Certainty Rating |
| --- | --- |
| Age (per year) | High |
| Age > (65 years) | High |
| Carotid artery stenosis | High |
| Diabetes | High |
| Hypertension | Moderate |
| Left ventricular ejection fraction (%) | High |
| Preoperative depression | High |
| NYHA functional class III or IV | High |
| Preoperative mild cognitive impairment | High |
| Preoperative statins | High |
| Aortic cross clamp time (per minute) | High |
| ICU stay (per day) | High |
| Mechanical ventilation time (per hour) | High |
